# Biology of Vascular Endothelial Growth Factor C in the Morphogenesis of Lymphatic Vessels

**DOI:** 10.3389/fbioe.2018.00007

**Published:** 2018-02-12

**Authors:** Khushbu Rauniyar, Sawan Kumar Jha, Michael Jeltsch

**Affiliations:** ^1^Translational Cancer Biology Research Program, University of Helsinki, Helsinki, Finland; ^2^Wihuri Research Institute, Biomedicum Helsinki, Helsinki, Finland

**Keywords:** vascular endothelial growth factor C, lymphatic vessels, lymphedema, tissue engineering, A disintegrin and metalloproteinase with thrombospondin motifs 3, collagen and calcium binding EGF domains 1, growth factors, VEGF receptors

## Abstract

Because virtually all tissues contain blood vessels, the importance of hemevascularization has been long recognized in regenerative medicine and tissue engineering. However, the lymphatic vasculature has only recently become a subject of interest. Central to the task of growing a lymphatic network are lymphatic endothelial cells (LECs), which constitute the innermost layer of all lymphatic vessels. The central molecule that directs proliferation and migration of LECs during embryogenesis is vascular endothelial growth factor C (VEGF-C). VEGF-C is therefore an important ingredient for LEC culture and attempts to (re)generate lymphatic vessels and networks. During its biosynthesis VEGF-C undergoes a stepwise proteolytic processing, during which its properties and affinities for its interaction partners change. Many of these fundamental aspects of VEGF-C biosynthesis have only recently been uncovered. So far, most—if not all—applications of VEGF-C do not discriminate between different forms of VEGF-C. However, for lymphatic regeneration and engineering purposes, it appears mandatory to understand these differences, since they relate, e.g., to important aspects such as biodistribution and receptor activation potential. In this review, we discuss the molecular biology of VEGF-C as it relates to the growth of LECs and lymphatic vessels. However, the properties of VEGF-C are similarly relevant for the cardiovascular system, since both old and recent data show that VEGF-C can have a profound effect on the blood vasculature.

## Introduction

Lymphatic endothelial cells (LECs) form the innermost layer of lymphatic vessels, and they play a central role during the growth of the lymphatic system (Bautch and Caron, 2015). Lymphatic insufficiency can be the result of an underdeveloped lymphatic network (Butler et al., 2009), and hence the idea of growing lymphatic structures has been proposed early on as a potential treatment strategy for lymphedema (Karkkainen et al., 2001a; Saaristo et al., 2002). Irrespective of whether these structures are regrown *in situ* (Karkkainen et al., 2001b; Dai et al., 2010; Moriondo et al., 2010; Güç et al., 2017) or *in vitro* (Helm et al., 2007; Gibot et al., 2017; Knezevic et al., 2017), the growth and assembly of LECs into vessels and networks are central to the task of lymphatic engineering (Kanapathy et al., 2014; Schaupper et al., 2016).

Therefore, it is appropriate—when setting out to (re)construct lymphatic vessels—to get familiar with LEC proliferation, migration, assembly, and maintenance. The central growth factor that mediates these tasks is vascular endothelial growth factor C (VEGF-C). While being a member of the VEGF family of growth factors, VEGF-C is in many aspects very different from the vascular endothelial growth factor prototype VEGF-A.

## VEGFs and VEGF Receptors (VEGFRs)

The primary receptors of all VEGF family members are tyrosine kinase receptors. With certain exceptions (Olsson et al., 2006), they are only expressed by endothelial cells. However, all three VEGFRs (VEGFR-1, -2 and -3) are not equally distributed on endothelial cells. In the adult organism, VEGFR-3 expression is largely restricted to LECs (Kaipainen et al., 1995), while VEGFR-1 expression is very low on LECs (Shibuya, 2001), and VEGFR-2 can be found both on LECs and blood vascular endothelial cells (BECs) (Holmes et al., 2007). VEGFRs are activated by dimerization, which is achieved by the dimeric nature of the VEGF ligands. The two receptor binding epitopes of each VEGF ligand are composite epitopes and are absent in monomeric VEGF species (Muller et al., 1997). Hence, monomeric VEGF species bind their respective receptors only with low affinity (Fuh et al., 1998) or not at all (Grunewald et al., 2010).

Apart from the VEGFRs, most VEGFs bind to co-receptors, which stabilize the VEGF/VEGFR interaction and increase the effective growth factor concentration on the cell surface, for example, neuropilins (Grunewald et al., 2010), integrins (Soldi et al., 1999), or syndecans (Johns et al., 2016). However, these interactions are typically of lower affinity than the VEGF/VEGFR interaction (Soker et al., 2002).

**Figure 1:**
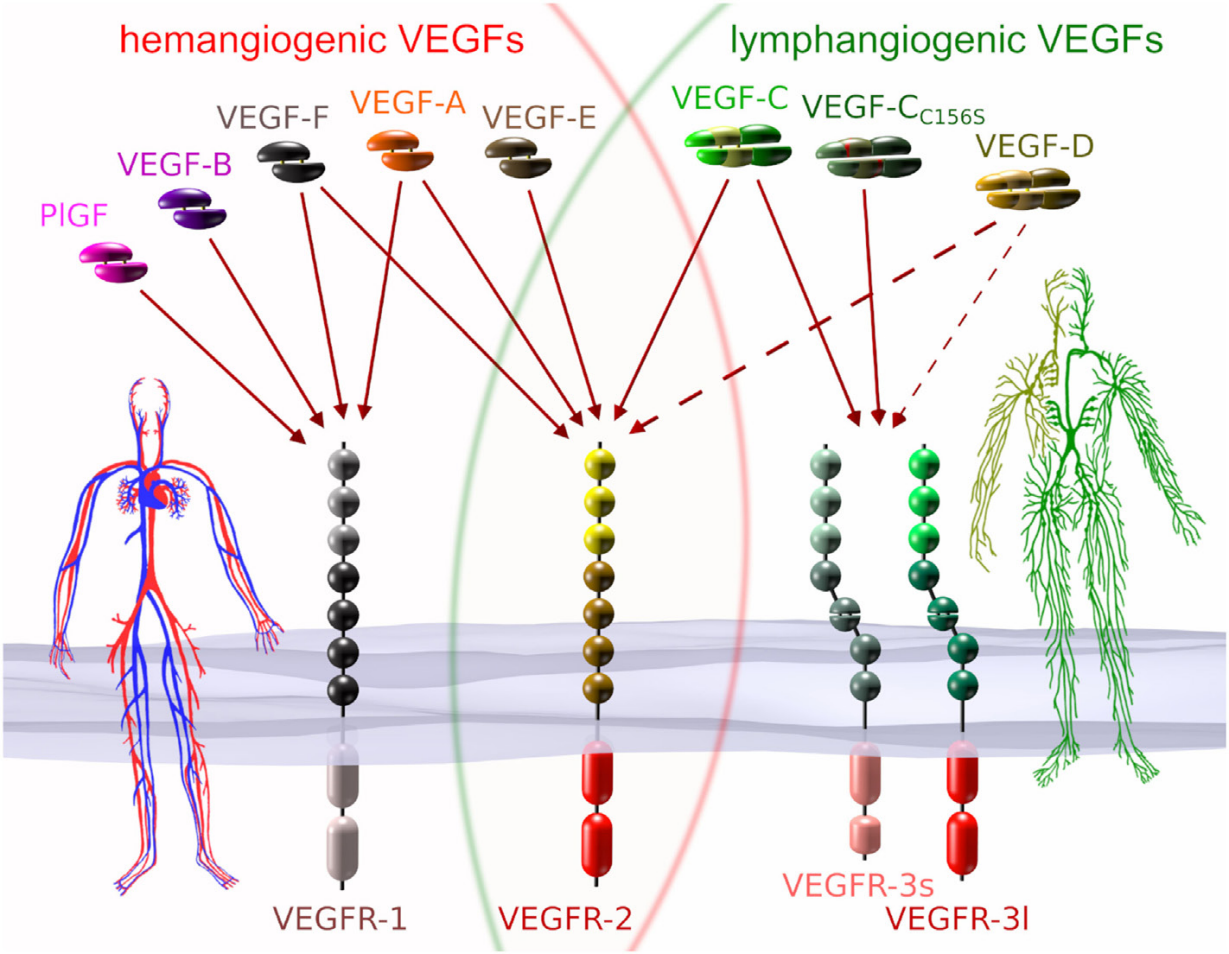
VEGFs and VEGF receptors (VEGFRs). Each of the five mammalian VEGFs [PlGF, VEGF-A to -D], the viral VEGF-E, and the snake venom VEGF-F interacts specifically with a certain subset of the three VEGFRs. VEGF-C_C156S_ is an engineered vascular endothelial growth factor C (VEGF-C) variant that interacts predominantly with VEGF receptor 3 (VEGFR-3) (Joukov et al., 1998). VEGFs that interact with all three receptors do not naturally exist, but have been engineered (Jeltsch et al., 2006). VEGF receptor 1 (VEGFR-1) and VEGF receptor 2 (VEGFR-2) are expressed on blood vascular endothelial cells (BECs), while VEGFR-2 and VEGFR-3 are expressed on lymphatic endothelial cells. VEGFR-3 is the primary mitogenic receptor for lymphatic endothelium, while VEGFR-2 is the primary mitogenic receptor for blood vascular endothelium. Exclusive to higher primates is the appearance of a short splice isoform of VEGFR-3 (VEGFR-3s) (Pajusola et al., 1993; Borg et al., 1995; Hughes, 2001). Signaling pathways activated by VEGFR-3s are partially distinct from those activated by the long splice isoform (VEGFR-3l), since it lacks some of the phosphorylation sites required for mediator docking (e.g., for Shc-Grb2) (Fournier et al., 1995; Dixelius et al., 2003). The dotted arrows from VEGF-D indicate heterogeneous binding patterns. While mature human VEGF-D can activate VEGFR-2, this seems not to be the case for mouse VEGF-D (Baldwin et al., 2001), and consequently, VEGF-D function could have diverged since the evolutionary divide some 60–65 million years ago (O’Leary et al., 2013). Additionally, human VEGF-D can selectively lose its affinity for VEGFR-3 after proteolytic processing (Leppanen et al., 2011).

In humans, five different genes encode VEGF family members: *VEGFA, VEGFB, VEGFC, VEGFD*, and *PGF* (placenta growth factor), respectively. Each VEGF can be roughly categorized as being hemangiogenic (VEGF-A, PlGF, and VEGF-B) or lymphangiogenic (VEGF-C and VEGF-D). Unique to the hemangiogenic VEGFs is their interaction with VEGFR-1, while only members of the lymphangiogenic group do interact with VEGFR-3. VEGFR-2, which is the receptor that drives proliferation and migration of BECs, can be activated by some but not all members from both groups (see Figure 1).

**Figure 2:**
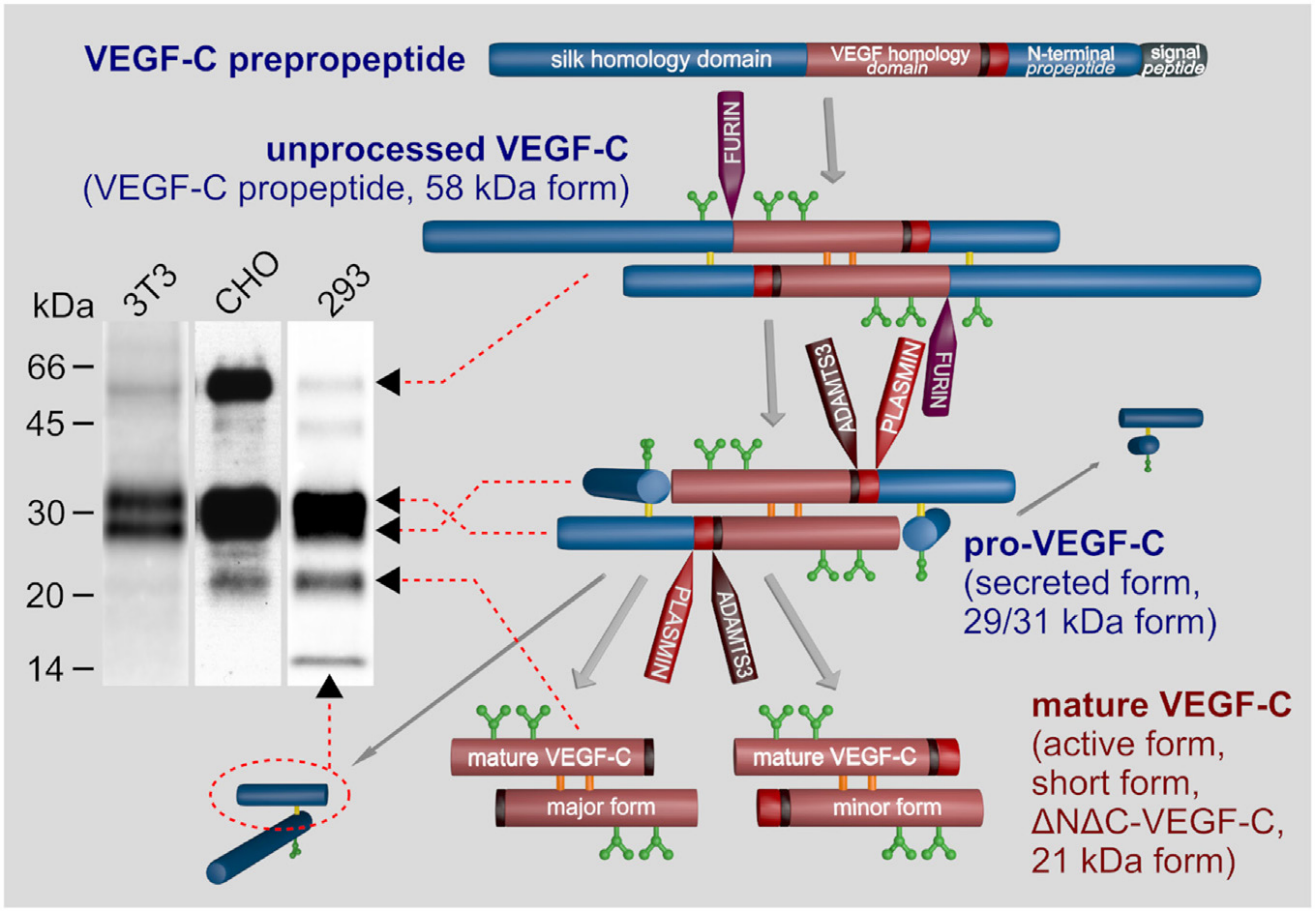
Biosynthesis and activation of vascular endothelial growth factor C (VEGF-C). VEGF-C is produced as an inactive propeptide. Proprotein convertases such as furin, PC5, or PC7 cleave between the VEGF homology domain and the C-terminal silk homology domain resulting in pro-VEGF-C. The silk homology domain is not removed by this cleavage, but remains covalently connected *via* cysteine bridges to the rest of pro-VEGF-C (Joukov et al., 1997b). Pro-VEGF-C is able to bind VEGFR-3, but does not activate it (Jeltsch et al., 2014). The second proteolytic cleavage by A disintegrin and metalloproteinase with thrombospondin motifs 3 (ADAMTS3) removes both terminal domains resulting in mature, active VEGF-C. Cleavage by ADAMTS3 results in the major form of the mature VEGF-C, which is nine amino acids shorter compared to the minor form, which is likely a product of plasmin cleavage (Joukov et al., 1998; Baldwin et al., 2001; Jeltsch et al., 2014). Three N-glycosylation sites are found in VEGF-C (shown in green). Alternative names for different VEGF-C forms are given in brackets. The band pattern of VEGF-C produced from a full-length cDNA resolved by SDS-PAGE depends on the expressing cell line, expression levels and the antibody used for immunoprecipitation and/or Western blotting. 3T3 fibroblasts produce almost exclusively pro-VEGF-C. In high-level-expressing CHO cells, a significant fraction of the secreted protein can remain unprocessed. Among the most efficiently processing cells are 293 cells, but pro-VEGF-C still represents the majority of the VEGF-C protein.

All VEGF family members are characterized by the central VEGF homology domain (VHD, aka PDGF/VEGF domain) (EMBL-EBI, 2017). The VHD contains the receptor binding domain and features a pattern of characteristically spaced cysteine residues, which gives rise to a cystine knot (Holmes and Zachary, 2005). In addition to the VHD, most VEGFs feature accessory sequences that further delineate the specific properties of individual VEGFs: the affinity of, e.g., VEGF-A to the co-receptors neuropilin-1 and -2 (NP-1 and NP-2) (Neufeld et al., 2002), or of VEGF-C to the co-receptor NP-2 (Karpanen et al., 2006; Xu et al., 2010), heparan sulfate proteoglycans (HSPGs) (Johns et al., 2016), and to the extracellular matrix (Jha et al., 2017). In VEGF-C and VEGF-D, the N- and C-terminal accessory sequences are exceptionally long and function as propeptides, which fold into own domains (see Figure 2) and need to be removed for activation by two proteolytic cleavages.

While only five genes encode the mammalian VEGFs, the actual number of different VEGFs is much larger. Within the hemangiogenic VEGFs, functional diversity is generated mostly by alternative splicing, resulting in differences in the affinity for HSPGs (“heparin affinity”) (Robinson and Stringer, 2001). For VEGF-C, no functions have been assigned yet to the described alternative splice variants (Lee et al., 1996; Ensembl, 2016).

## The Lymphangiogenic VEGFs

Vascular endothelial growth factor C was discovered more than 20 years ago as a binding partner of VEGFR-3 from the cell culture supernatant of the human prostate cancer cell line PC3 (Joukov et al., 1996). In the same year, also murine VEGF-C was described and initially named VRP (VEGF-related protein) (Lee et al., 1996). The specific lymphangiogenic properties of VEGF-C were demonstrated in various animal models (Jeltsch et al., 1997; Oh et al., 1997). VEGF-D is the second member of the lymphangiogenic VEGF subgroup. It was discovered independently by three research teams and named once FIGF (c-fos-induced growth factor) (Orlandini et al., 1996) and twice VEGF-D (Yamada et al., 1997; Achen et al., 1998). Both VEGF-C and VEGF-D use posttranslational modification by proteolytic cleavage to generate molecular species diversity (Joukov et al., 1997b; Stacker et al., 1999), but the proteases are different (Bui et al., 2016). While VEGF-D shares many similarities with VEGF-C, it cannot replace VEGF-C (see the VEGF-D paragraph).

## Different VEGF-C Forms, the VEGF-C_C156S_ Mutant and Angiogenic “Side Effects”

The most prominent differences between the different VEGF-C forms are the affinities for the receptors, co-receptors, and the extracellular matrix. With increasing processing (see Figure 2), VEGF-C’s affinity for both VEGFR-2 and VEGFR-3 increases, and fully processed mature VEGF-C is therefore not only lymphangiogenic but also angiogenic (Joukov et al., 1997a; Anisimov et al., 2009) and induces the permeability of blood vessels (Joukov et al., 1997b). To identify which functions of VEGF-C are mediated by which receptor (VEGFR-2 versus VEGFR-3), the VEGF-C_C156S_ mutant was developed (Joukov et al., 1998) (commercially available from R&D systems as 752-VC or its rat homolog from Reliatech as R20-016). This mutant largely lacks VEGFR-2 affinity and can therefore be used to exclude VEGF-C effects on the blood vasculature (Veikkola et al., 2001). However, despite its dual receptor binding, the effect of wild type VEGF-C on the blood vasculature is minimal in many models, and recent data suggest that the localization of pro-VEGF-C on LEC surfaces prior to activation could explain this specificity (Jha et al., 2017). Most reports that attribute a prominent angiogenic effect to VEGF-C have investigated prenatal development (Oh et al., 1997; Lohela et al., 2008) or used VEGF-C forms lacking the propeptides (Oh et al., 1997; Cao et al., 1998; Sweat et al., 2014). The propeptides keep VEGF-C inactive (Joukov et al., 1997b), are required for localizing VEGF-C (Jha et al., 2017), and are removed by sequential proteolysis by furin (Siegfried et al., 2003) and A disintegrin and metalloproteinase with thrombospondin motifs 3 (ADAMTS3) (Jeltsch et al., 2014) or plasmin (McColl et al., 2003).

The relationship between affinity and receptor activation is not straightforward: pro-VEGF-C is able to bind VEGFR-3 under cooperation of NP-2 without any detectable receptor activation. In fact, it is even a competitive inhibitor of mature VEGF-C for VEGFR-3 activation (Jeltsch et al., 2014).

## VEGF-D

VEGF-D is the closest paralog of VEGF-C (Achen et al., 1998). The angiogenic potential of mature VEGF-D has been shown to be stronger compared to mature VEGF-C (Rissanen et al., 2003), which is explained by the fact that maximally processed VEGF-D exclusively binds to VEGFR-2, while VEGF-C retains in its maximally processed form the capacity to bind VEGFR-3 (Leppanen et al., 2011). It is differently activated than VEGF-C (Bui et al., 2016), and because the proteolytic environment is difficult to predict and control, the use of VEGF-D for LEC stimulation remains problematic. Moreover, *Vegfd* gene-deleted mice have no lymphatic phenotype arguing for no major role in the development of the murine lymphatic system (Baldwin et al., 2005).

## Requirement for Collagen and Calcium Binding EGF Domains 1 (CCBE1) AND ADAMTS3

The two molecules that are required for the proteolytic activation of VEGF-C are the CCBE1 protein and the ADAMTS3 protease. ADAMTS3 catalyzes the final step in the proteolytic processing of VEGF-C, removing the N-terminal propeptide and releasing the fully active, mature VEGF-C (Jeltsch et al., 2014; see Figure 2). *In vitro*, large amounts of ADAMTS3 are able to activate pro-VEGF-C, but *in vivo*, ADAMTS3 requires the assistance of CCBE1 for the efficient activation of VEGF-C. CCBE1 enhances the VEGF-C activation by two different mechanisms: it increases the processivity of the ADAMTS3 enzyme (Roukens et al., 2015) and it colocalizes VEGF-C and ADAMTS3 on cell surfaces and ECM to form the trimeric activation complex (Bui et al., 2016; Jha et al., 2017). Similar to *Vegfc*, the genetic ablation of either *Ccbe1* or *Adamts3* in mice results in a general halt of lymphatic development (Bos et al., 2011; Hagerling et al., 2013; Janssen et al., 2015).

A disintegrin and metalloproteinase with thrombospondin motifs 3 cleavage results in the so-called “major” form of mature VEGF-C (Joukov et al., 1998; Jeltsch et al., 2014). The so-called “minor” form is nine amino acids longer at its N-terminus and is presumably generated by plasmin. Plasmin might activate VEGF-C during wound healing and inflammatory processes (McColl et al., 2003), where it could rapidly release large amounts of active VEGF-C from matrix-bound, “latent” pro-VEGF-C. However, it is still unclear, whether there is any difference between the lymphangiogenesis response to the “major” and “minor” forms of mature VEGF-C.

**Figure 3:**
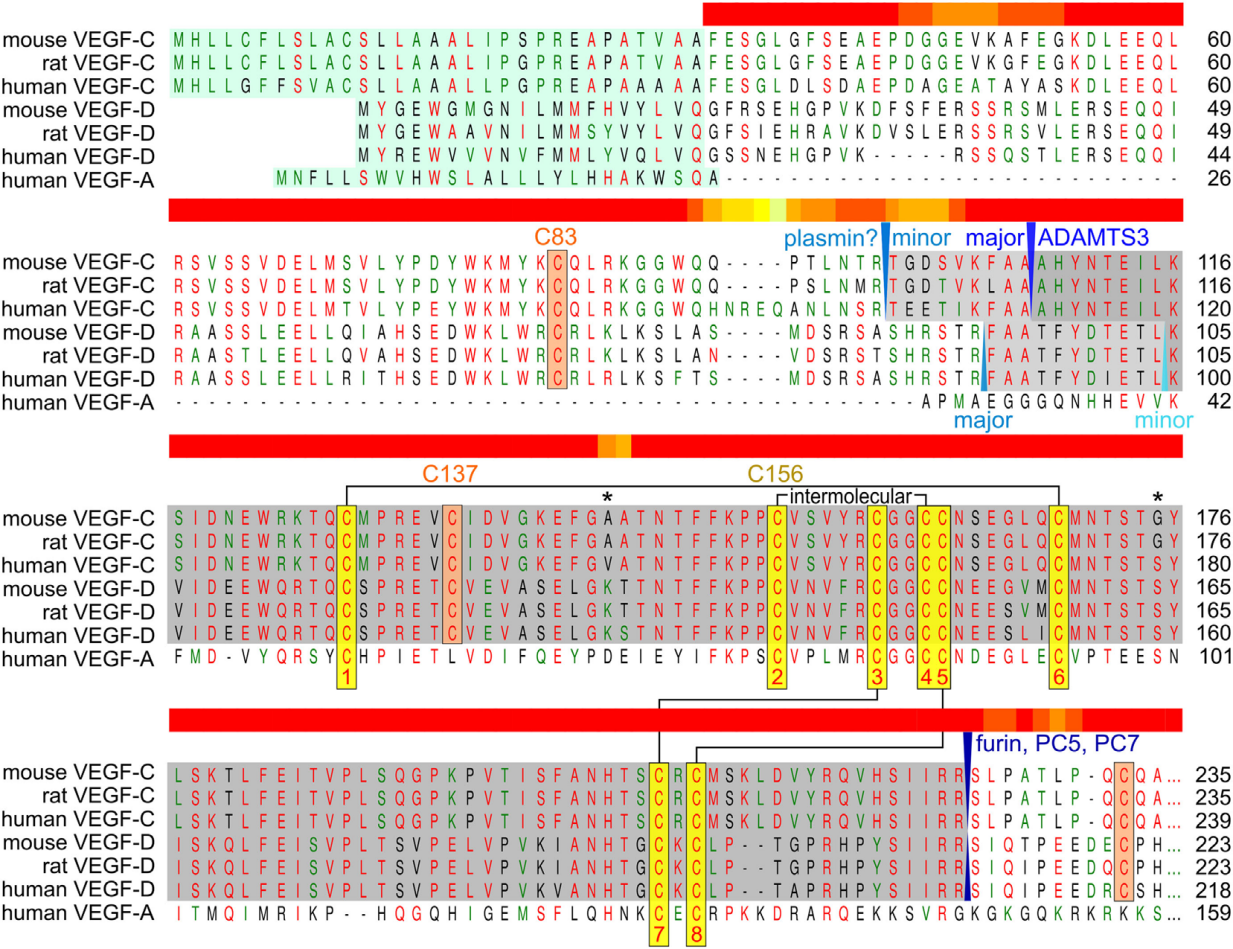
Alignment of human, mouse, and rat vascular endothelial growth factor C (VEGF-C)/D with human VEGF-A. The sequences of the active, mature VEGF-C/D are boxed gray. Proteolytic cleavage sites and enzymes (if known) are indicated in blue colors. The signal peptide is boxed green. The eight conserved cysteines of the PDGF/VEGF signature (Muller et al., 1997) are boxed yellow and intra- and intermolecular disulfide bridges are indicated by black connecting lines. VEGF-C/D-specific conserved cysteine residues are boxed in orange. The two asterisks denote the only two amino acid residues that are different between fully processed mouse and human VEGF-C. Cysteine 156, which is mutated to serine in the VEGFR-3-monospecific variant VEGF-C_C156S_, participates in the intermolecular cystine bridge (Joukov et al., 1998). When mature VEGF-C is produced from a truncated cDNA, the single cysteine 137 remains unpaired decreasing protein stability (Anisimov et al., 2009; Leppanen et al., 2011). When pairing with cysteine 156, cysteine 137 interferes with intermolecular disulfide bond formation and protein folding, explaining the observation of significant amounts of single-linked dimers, non-covalent VEGF-C dimers and VEGF-C monomers (Joukov et al., 1996; Jeltsch et al., 2006; Chiu et al., 2014). Above the alignment, a heat map indicates the areas of highest divergence, deduced from a more comprehensive alignment of VEGF-A, -C, and -D. The C-terminal domains of VEGF-C/D are not shown.

To bypass the complex proteolytic maturation of VEGF-C, the recombinant production of mature VEGF-C is almost exclusively done from a truncated cDNA. However, this is not without problems, since VEGF-C contains in its VHD—as opposed to VEGF-A—an extra cysteine residue (Cys 137), which interferes with intermolecular disulfide bond formation (Chiu et al., 2014) and protein stability (Anisimov et al., 2009; Leppanen et al., 2011) (see Figure 3). It has been proposed that when produced from a full-length cDNA, cysteine residues from the VEGF-C propeptides could protect Cys 137 and thus facilitate correct disulfide bond formation in the VHD.

## The Enigmatic “Silk Homology” Domain

Very intriguing is the repetitive arrangement of cysteine residues (CX_10_CXCXC) in the C-terminal propeptide of VEGF-C. This signature is unique within vertebrate proteins, and its phylogenetic origin remains unknown. Except in VEGF-C, it occurs, e.g., in the balbiani ring protein 3 and salivary proteins of silk weaving mosquito larvae of the genus Chironomus (Dignam and Case, 1990). Therefore, the term “silk homology domain” was coined to describe the C-terminal propeptide (Joukov et al., 1996). In addition to regulating the activity of VEGF-C, this domain endows the molecule with most of its heparin affinity (Johns et al., 2016), and it is the determining factor for the ECM sequestration of VEGF-C (Jha et al., 2017). Interestingly, sequestration and proteolysis-mediated release of active VEGF-C have also been reverse engineered by concatenating a fibrin-binding (FB) and a MMP-degradable polypeptide sequence N-terminally to the mature VEGF-C sequence (Güç et al., 2017). This protein (FB-VEGF-C) compared favorably to mature VEGF-C in the local induction of lymphangiogenesis, but native sequestration of wild-type VEGF-C expressed from a full-length cDNA was not included in this comparison.

## Regulation of VEGF-C Signaling

Compared to VEGF-A, not much is known about the regulation of VEGF-C expression *in vivo*. The fact that VEGF-C is produced as an inactive precursor (pro-VEGF-C) indicates that much of its regulation happens after its constitutive secretion at the level of proteolytic activation. However, while the activation mechanism itself has been studied extensively (Jeltsch et al., 2014; Roukens et al., 2015; Bui et al., 2016; Jha et al., 2017), virtually nothing is known about the regulation of the obligatory protease ADAMTS3 and its cofactor CCBE1.

In the adult organism, inflammation potently upregulates VEGF-C expression, most notably by macrophages (Ristimäki et al., 1998; Baluk et al., 2005; Krebs et al., 2012), resulting in a negative feedback loop (Zhou et al., 2011; Christiansen et al., 2016) promoting resolution in some models, while aggravating the situation in others (Kim et al., 2014).

The interstitial pressure that builds up during development in blood-vascularized but lymphatic-free tissues leads *via* β1-integrin-mediated mechanoinduction to enhanced VEGFR-3 phosphorylation (Planas-Paz et al., 2012). However, it remains unclear, whether meaningful VEGFR-3 phosphorylation can happen *in vivo* entirely without ligand, although the kinase activity of VEGFR-3 is dispensable *in vitro* (Galvagni et al., 2010). In any case, the interplay of mechanical forces with growth factor signaling for the establishment of functional lymphatic networks has been shown in many systems (Sabine et al., 2016). Binding of mature VEGF-C to VEGFR-3 results in the activation of both the MAPK/ERK and AKT intracellular signaling pathways (Mäkinen et al., 2001; Deng et al., 2015), which promote survival, growth, and migration in LECs (Mäkinen et al., 2001).

## VEGF-C Signaling in Embryonic Development

VEGF receptor 3 was discovered before VEGF-C and VEGF-D, and therefore, VEGFR-3 was between 1992 and 1996 an “orphan receptor”, i.e., a receptor without known ligand. However, soon after the discovery of VEGFR-3, the specific expression pattern of VEGFR-3 suggested that its function was closely related to the lymphatic system. In the early stages of embryonic development, all endothelial cells express VEGFR-3, but its expression becomes progressively restricted to LECs (Kaipainen et al., 1995). Finally, VEGFR-3 expression becomes sufficiently specific for LECs that it has been used to identify LECs (Petrova et al., 2008), despite the existence of other VEGFR-3 expressing endothelial cells, e.g., angiogenic, sinusoidal, and fenestrated BECs.

The pivotal role of the VEGFR-3 ligand VEGF-C in the establishment of the lymphatic vasculature is witnessed by the fact that mice devoid of VEGF-C do not develop any lymphatic structures and form generalized edema from E12.5, resulting in embryonic death around E16.5 (Karkkainen et al., 2004).

Interestingly, mice devoid of the VEGF-C receptor VEGFR-3 die already around E9.5, before the first lymphatic structures develop, from failures in the organization and maturation of blood vessels (Dumont et al., 1998). However, embryonic lethality after deletion of both VEGFR-3 ligands, VEGF-C and VEGF-D, occurs only around E16.5, suggesting no role of these ligands for VEGFR-3 activation in early embryogenesis (Haiko et al., 2008). Since VEGFR-3 can form heterodimers with VEGFR-2, this might be a substitute mechanism for VEGFR-3 activation (Dixelius et al., 2003; Nilsson et al., 2010). Alternatively, ligandless baseline signaling (Zhang et al., 2005; Galvagni et al., 2010), mechanoinduction (Planas-Paz et al., 2012), or unrecognized ligands might provide a sufficient stimulus. Interestingly, neither the development of the blood vascular system nor of the lymphatic vascular system is affected by the lack of the second lymphangiogenic growth factor VEGF-D (Baldwin et al., 2005).

## Genetic Lesions in the VEGF-C/VEGFR-3 Signaling Pathway

So far, mutations in 27 genes have been found to cause human lymphedema conditions. Several of these mutations affect components of the VEGF-C/VEGFR-3 signaling pathway (Table 1). Interestingly, a major fraction of hereditary lymphedema patients present with mutations in *VEGFR-3*, while mutations in the other genes are relatively rare (Brouillard et al., 2014, 2017).

**Table 1:** Hereditary human lymphedema conditions involving the vascular endothelial growth factor C (VEGF-C)/VEGF receptor 3 (VEGFR-3) signaling pathway.

*GENE* (protein)	Human condition (OMIM, alternative name)	Lymphedema phenotype	Reference for the initial linkage	Molecular etiology	Viable animal models
*FLT4* (VEGFR-3)	Hereditary lymphedema type 1A (153100, Milroy disease)	Predominantly the lower extremities	Irrthum et al. (2000), Karkkainen et al. (2000)	Dominant negative inactivation of the intracellular kinase domain (Irrthum et al., 2000; Karkkainen et al., 2000)	Chy mice (inactivating *Flt4* mutation) (Karkkainen et al., 2001b); VEGFR-3 inhibition (Mäkinen et al., 2001)
	
*VEGFC* (VEGF-C)	Hereditary lymphedema type 1D (615907, Milroy-like disease)		Gordon et al. (2013), Balboa-Beltran et al. (2014)	Secretion defect (Gordon et al., 2013; Villefranc et al., 2013)	Chy-3 mice (hemizygous *Vegfc* deletion) (Dellinger et al., 2007); conditional *Vegfc* ko mice (Nurmi et al., 2015)

*CCBE1* (Collagen and calcium-binding EGF domain-containing protein 1)	Hennekam syndrome type 1 (235510)	Generalized	Alders et al. (2009)	A disintegrin and metalloproteinase with thrombospondin motifs 3 activation defect (Jeltsch et al., 2014; Roukens et al., 2015), localization defect (Jha et al., 2017)	Conditional *Ccbe1* ko mice (Bui et al., 2016)
	Cholestasis–lymphedema syndrome (214900, Aagenaes syndrome)		Shah et al. (2013), Viveiros et al. (2017)		
	
*FAT4* (Protocadherin Fat4)	Hennekam syndrome type 2 (616006)		Alders et al. (2014)	Unknown molecular etiology	Vascular abnormalities were not reported for the full *Fat4* ko mice (Saburi et al., 2008)
	Van Maldergem syndrome type 2 (615546)				
	
*ADAMTS3* (A disintegrin and metalloproteinase with thrombospondin motifs 3)	Hennekam syndrome type 3		Brouillard et al. (2017)	Secretion defect (Brouillard et al., 2017), localization defect (Jha et al., 2017)	Conditional *Adamts3* ko mice (Bui et al., 2016)

Although in hereditary lymphedema type 1A and 1D, all cells carry the mutant *VEGFR-3* or *VEGF-C* gene, not all lymphatic vessels and body parts are equally affected. Hypoplastic or aplastic lymph capillaries are mainly found in peripheral, superficial regions (Bollinger et al., 1983). However, in addition to the underdeveloped lymphatic structures, a functional deficit seems to play a variable, but significant role in the manifestation of the edema (Mellor et al., 2010). A higher hydrostatic pressure resulting in increased drainage needs in the extremities could possibly explain localized symptoms. However, leg edema is as well observed in mice and newborns, where hydrostatic pressure differences are negligible (Karkkainen et al., 2001a). Alternatively, LECs from different vascular beds could have a different sensitivity for VEGFR-3 signaling, perhaps due to a different developmental origin.

## LEC Culture and VEGF-C Signaling

During embryonic growth, signaling by VEGF-C is necessary for the establishment of the lymphatic vasculature (Karkkainen et al., 2004). In the adult organism, the dependency on VEGF-C is less pronounced, and ablation of VEGF-C in the adult organism appears well tolerated also over longer periods of time, except for the intestinal lymphatics (the lacteals) and meningeal lymphatics, which depend on a steady supply with VEGF-C (Nurmi et al., 2015; Antila et al., 2017). It is not known, whether this difference is due to the higher stress or increased turnover of intestinal LECs compared to, e.g., adult skin LECs, which are mostly in the resting phase (Alexander et al., 2010). Despite the apparent VEGF-C requirements for proliferating LECs *in vivo*, LEC vendors specifically endorse only the use of the hemangiogenic VEGF-A for LEC culture. However, some researchers have modified such media to contain VEGF-C (Mäkinen et al., 2001; Petrova et al., 2002; Podgrabinska et al., 2002; Veikkola et al., 2003). While serum does typically contain VEGF-C in the single digit ng/ml-range (R&D Systems, 2017), serum-supplemented LEC culture medium would still contain only small amounts of VEGF-C compared to VEGF-A, which stimulates VEGFR-2, but not VEGFR-3. While VEGF-A stimulates LEC proliferation and lymphatic vessel dilation *in vitro* (Dellinger and Brekken, 2011) and increases the density of lymphatic *in vitro* capillary networks (Marino et al., 2014) to a similar degree as VEGF-C, its importance for *in vivo* LEC proliferation is arguable as only very few *in vivo* models of VEGF-A application seem to directly affect lymphatic networks (Shin et al., 2008). While VEGFR-2 and VEGFR-3 activation both result in PKC-dependent Akt phosphorylation, the activation routes and kinetics differ (Mäkinen et al., 2001). Importantly, SOX18 and KLF4, which are implicated in LEC differentiation (Francois et al., 2008; Park et al., 2014), are specifically regulated by VEGFR-3 (Dieterich et al., 2017).

The specific form of VEGF-C that is used for LEC culture supplementation is the active, mature form, but pro-VEGF-C also might be an attractive option, since the LEC-expressed ADAMTS3 and CCBE1 (Jha et al., 2017) would concertedly convert it into the mature form, providing a differently localized and perhaps more sustained stimulus. Due to its potent synergistic effect with VEGF-C, hepatocyte growth factor (HGF) also needs consideration as a LEC culture additive (Kajiya et al., 2005; Gibot et al., 2016).

## When VEGF-C is Not Enough

While in some models, the angiogenic component of VEGF-C can be exposed (e.g., in the heart) (Losordo et al., 2002; Chen et al., 2014), VEGF-C predominantly affects the lymphatic system. Not surprisingly, most therapeutic applications of VEGF-C are targeting the lymphatics (Tammela et al., 2007; Honkonen et al., 2013; Klotz et al., 2015), and VEGF-C should therefore be considered the primary growth factor of choice in lymphatic engineering tasks that involve LECs. While necessary, VEGF-C alone is not sufficient for the successful establishment of a functional lymphatic network in some situations (Goldman et al., 2005), and blocking signals that inhibit lymphangiogenesis such as TGF-β1 (Avraham et al., 2010) might be necessary. An elegant way bypassing the need for VEGF-C supplementation is the coculture of LECs with other cell types. In addition to secreted factors, cell–cell contacts appear important for the establishment of lymphatic networks. In one model, lymphangiogenesis was sustained by fibroblast-derived VEGF-C and HGF. While VEGF-C appeared to be constitutively expressed by fibroblasts, HGF expression was induced only in the cocultures (Gibot et al., 2016). Whether the fibroblasts were also able to stimulate the release of Reelin from the LECs was not analyzed in this model. Reelin release from LECs is normally induced by smooth muscle cell contacts and is required for the establishment of collecting vessels (Lutter et al., 2012). In another coculture model, LECs, BECs, and adipose-derived stromal cells (ASCs) in a 3D fibrin matrix depended on the addition of exogenous VEGF-C for substantial LEC network formation in addition to cell–cell contacts between LECs and ASCs (Knezevic et al., 2017). In the same model, BEC network formation was not affected by the absence of exogenous VEGF-C.

## Directing Regenerative Lymphatic Growth *In Vivo*

The mechanisms of the directional growth are similar for blood vessels and nerve cell axons (Carmeliet, 2003): a specialized cell on the tip of the vascular sprout (tip cell) determines the direction of growth of subsequent cells (stalk cells) by extending filopodia with growth factor receptors (Gerhardt et al., 2003). However, *in vivo* evidence for the importance of VEGF gradients for the directed growth of vascular networks is sparse (Ruhrberg et al., 2002), and also *in vitro*, convincing evidence is largely absent (Bautch, 2012). Likewise, filopodia seems to be dispensable for vascular patterning (Wacker et al., 2014). In the expansion of lymphatic networks, similar directed sprouting can be observed, but e.g. in the mouse tail lymphedema model, the mere application of VEGF-C was not enough to induce sprouting lymphangiogenesis (Goldman et al., 2005). Moreover, and contrary to expectations, VEGF-C levels correlated even in some models with lymphedema formation, apparently *via* inducing vascular leakage and immune cell infiltration (Gousopoulos et al., 2017). Surgical grafting of engineered small lymphatic structures is difficult unless they are grafted as part of a larger tissue (e.g., a vascularized skin graft). Hence, the idea of generating lymphatics *in situ* is attractive and indeed has been successfully achieved in some animal models using different delivery strategies for VEGF-C (Karkkainen et al., 2001b; Szuba et al., 2002; Yoon et al., 2003). The question whether VEGF-C alone is enough (Breier, 2005; Goldman et al., 2005) has recently at least received a partial answer by the discovery of obligatory cofactors such as CCBE1 and ADAMTS3 for correct VEGF-C localization and efficient activation (Jeltsch et al., 2014; Jha et al., 2017), and the presence or absence of these factors might explain differences in the lymphatic response. After encouraging preclinical studies (Tammela et al., 2007), the therapeutic value of VEGF-C for an improved integration of transplanted lymph nodes into the regional lymph system is currently under investigation (Tervala et al., 2015). It is less obvious how large collecting lymphatics could be generated *in situ*, but flow-stimulated remodeling of smaller lymphatics might happen akin to the hemodynamic remodeling of blood vessels (Culver and Dickinson, 2010).

**Figure 4:**
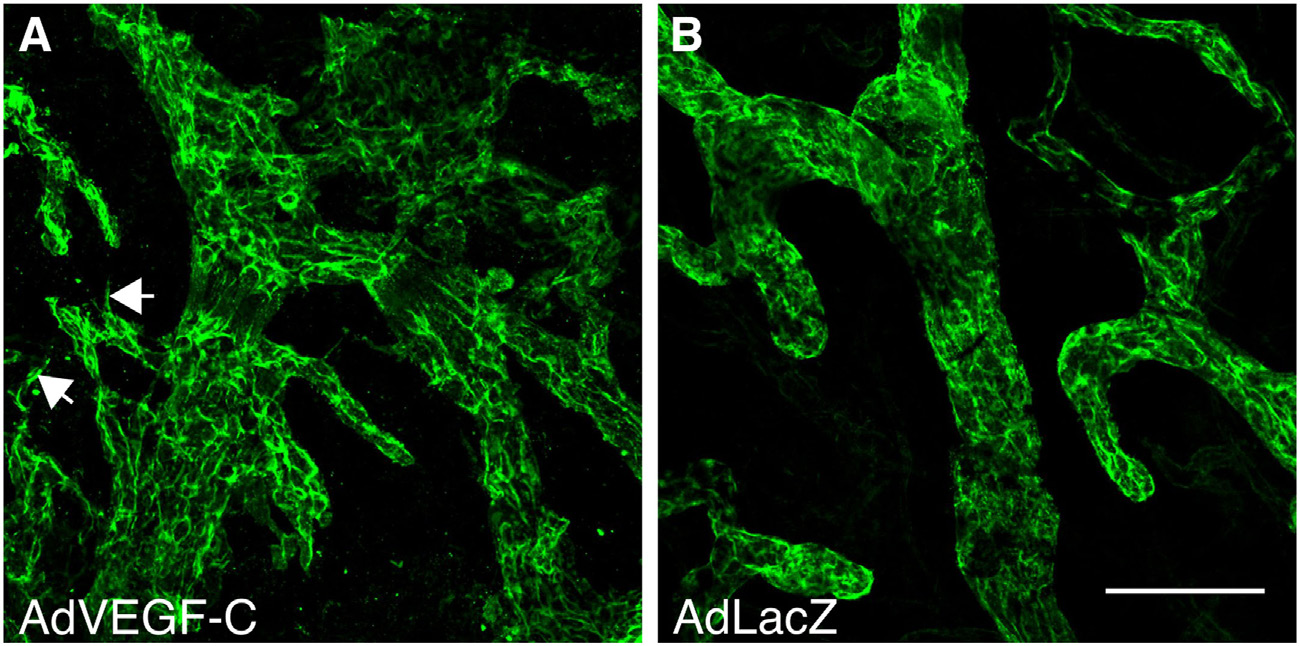
Vascular endothelial growth factor C (VEGF-C) induces specifically the growth of the lymphatic vasculature. Whole-mount LYVE-1 staining of mouse ears 2 weeks after adenoviral transduction with VEGF-C **(A)** and LacZ **(B)**. AdVEGF-C induces hyperplasia of and neo-sprouting from the lymphatic vasculature. Arrows indicate lymphatic sprouting. Bar, 100 µm.

**Figure 5:**
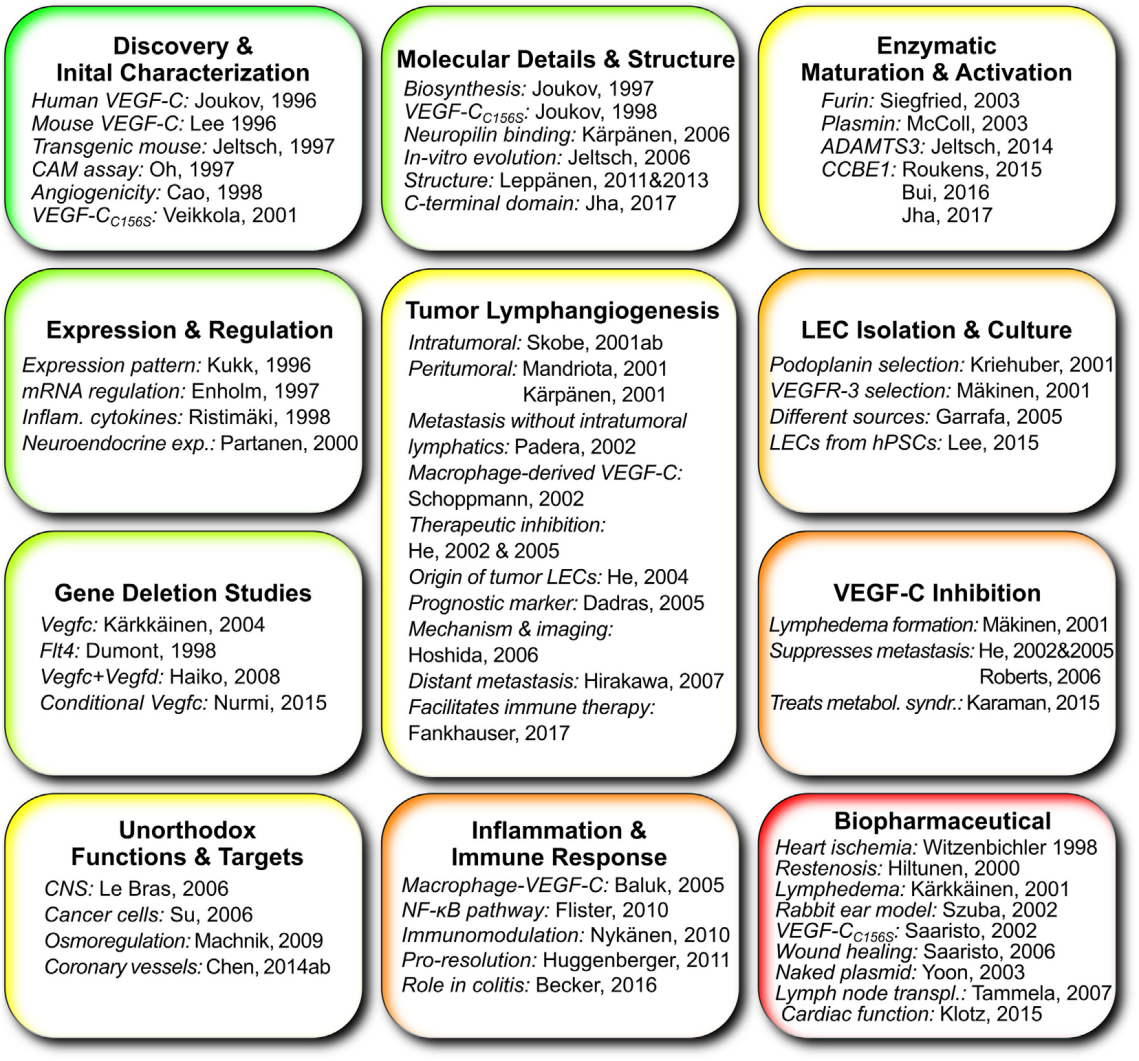
Works that give important insights into vascular endothelial growth factor C (VEGF-C) and its function grouped according to topic. Publications about the use of VEGF-C specifically in lymphatic tissue engineering are not included since this list tries to highlight the elemental scientific insights on which tissue engineering can build.

Although preclinical experience with *in situ* reconstitution of lymphatic networks using VEGF-C exists for more than 15 years, there is only one ongoing phase I clinical trial using VEGF-C therapy, namely the application of a VEGF-C-expressing adenovirus (AdVEGF-C, see Figure 4), in combination with lymph node transplantation for the treatment of secondary lymphedema after breast cancer surgery. The slow adoption might partly result from animal studies that identify VEGF-C as a key inducer for the growth and dissemination of certain tumors (for references, see Figure 5). While it is prudent to take the complete picture of VEGF-C biology as shown in Figure 5 into consideration when reconstructing lymphatics, the localized and limited availability of VEGF-C in this specific ongoing phase I study should exclude tumor-promoting side effects, paving the way for phase II studies.

## Author Contributions

All authors listed have made a substantial, direct, and intellectual contribution to the work, and approved it for publication.

## Conflict of Interest Statement

The authors declare that the research was conducted in the absence of any commercial or financial relationships that could be construed as a potential conflict of interest.
